# Comparison of reperfusion- and central repair-first strategies for acute type A dissection with mesenteric malperfusion: a single-center retrospective cohort study

**DOI:** 10.1097/JS9.0000000000001746

**Published:** 2024-05-28

**Authors:** Xia Gao, Yu-Xin Chen, Wei-Guo Ma, Li Zhang, Cong Cui, Ping-Fan Wang, Yi-Qiang Yuan

**Affiliations:** a Departments of Cardiovascular Surgery and Cardiology, Henan Chest Hospital, Zhengzhou University, Zhengzhou, China; b Division of Cardiac Surgery, Yale Medical School, New Haven, CT, USA

**Keywords:** mesenteric malperfusion/ischemia, operative mortality/morbidity, superior mesenteric stenting, survival, type A aortic dissection

## Abstract

**Background:**

We seek to compare the early and late outcomes of reperfusion-first vs. central repair-first strategies in patients with acute type A dissection (ATAAD) complicated by mesenteric malperfusion.

**Methods:**

Among 68 patients, reperfusion-first strategy with superior mesenteric artery (SMA) stenting was adopted in 31 and central repair-first in 37, based on rupture risk and circulatory compromise, severity, time and mechanisms of mesenteric ischemia. Early and late outcomes were compared between two strategies. Follow-up was 100% at 3.3±1.4 years.

**Results:**

Mean age was 50.6±11.4 years (59 males, 86.8%). The reperfusion-first group were more likely to have celiac artery involvement (74.2% vs. 48.6%, *P*=0.033) and peritoneal irritation signs (19.4% vs. 2.7%, *P*=0.025), while central repair-first group had more tamponade (27% vs. 3.2%, *P*=0.008). Early mortality was 48.6% (18/37) with central repair-first strategy vs. 19.4% (6/31) in reperfusion-first group (*P*=0.012). Reperfusion-first patients had fewer gastrointestinal complications (12.9% vs. 54.1%, *P*<0.001) and respiratory failure (3.2% vs. 24.3%, *P*=0.017). At 5 years, SMA stent patency was 84%, and survival was significantly higher in reperfusion-first patients (80.6% vs. 45.9%, *P*=0.009), with similar freedom from adverse events between two groups (74.9% vs. 76.0%, *P*=0.812). Tamponade [hazard ratio (HR), 3.093; *P*=0.023], peritoneal irritation signs (HR, 8.559; *P*=0.006), and lactate (mmol/l) (HR, 1.279; *P*<0.001) were predictors for all-cause mortality.

**Conclusions:**

In this series of ATAAD patients with mesenteric malperfusion, the reperfusion-first strategy with SMA stenting significantly reduced the mortality risk and achieved favorable late survival and freedom from adverse events. These results argue favorably for the use of the reperfusion-first strategy in acute type A dissection with mesenteric malperfusion.

Highlights

- This study may represent by far the largest cohort comparing the central repair-first vs. reperfusion-first strategies in the treatment of patients with acute type A dissection complicated by mesenteric malperfusion.
- Early mortality was 48.6% with the central repair-first strategy in this series.
- A reperfusion-first strategy with superior mesenteric artery stenting reduced early mortality by 60% (48.6% vs. 19.4%), and achieved favorable midterm survival and freedom from adverse events, with high stent patency.
- Triage algorithm based on aortic rupture risk and circulatory compromise, and the severity, duration and mechanisms of mesenteric ischemia may improve treatment outcomes of this highly lethal catastrophe.

Despite the advances in the diagnosis and management of acute type A aortic dissection (ATAAD)^1–3^, this catastrophic condition is still associated with significant mortality of 20–25% after surgery^4^. In patients with ATAAD complicated by malperfusion syndrome, the prognosis is even worse^5,6^. According to data from the International Registry of Acute Aortic Dissection (IRAD)^7^, the mortality rate was 29% for malperfusion syndrome, and 43% in those with ischemia of 3 or more organ/tissues, while mesenteric malperfusion was the most fatal, with a mortality as high as 41–69%^7–9^. Although emergency ATAAD repair is the accepted primary strategy, some centers advocate early reperfusion using fenestration or stenting to restore visceral perfusion and perform delayed central repair when the patient is stabilized^10,11^. However, the optimal treatment strategy for ATAAD with mesenteric malperfusion, that is central repair- vs. reperfusion-first, has yet to be established given the limited experience and lack of long-term evidence in large series^7,10,12–17^.

In our institution, we have been using both central repair-first and reperfusion-first strategies in the management of ATAAD with mesenteric malperfusion. This study aims to compare the early and late outcomes of two strategies in such patients over a 6-year period.

## Patients and methods

This work has been reported in line with the STROCSS criteria^18^.

### Patients

**Figure 1:**
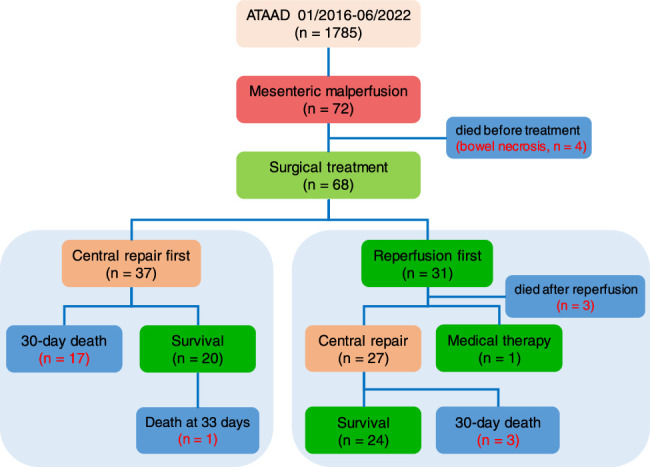
Flowchart of patient inclusion and treatment outcomes. ATAAD, acute type A aortic dissection.

Between 01/2016 and 06/2022, we have treated 1785 patients with ATAAD, 72 of whom (4.0%) were diagnosed with superior mesenteric artery (SMA) involvement. The diagnosis was made based on clinical presentation (abdominal pain/distension, hematochezia, changes in bowel sounds, and peritoneal irritation signs), computed tomography (CT) findings (reduced or no flow in SMA, with/without thrombosis), and laboratory tests (elevated lactate with/without acidosis). Four patients died of bowel necrosis preoperatively, and the 68 remaining were included in this study, who were divided into the reperfusion-first (*n*=31) and central repair-first groups (*n*=37) according to the prioritized treatment approach (Fig. 1).

### Patient triage and selection criteria

**Figure 2:**
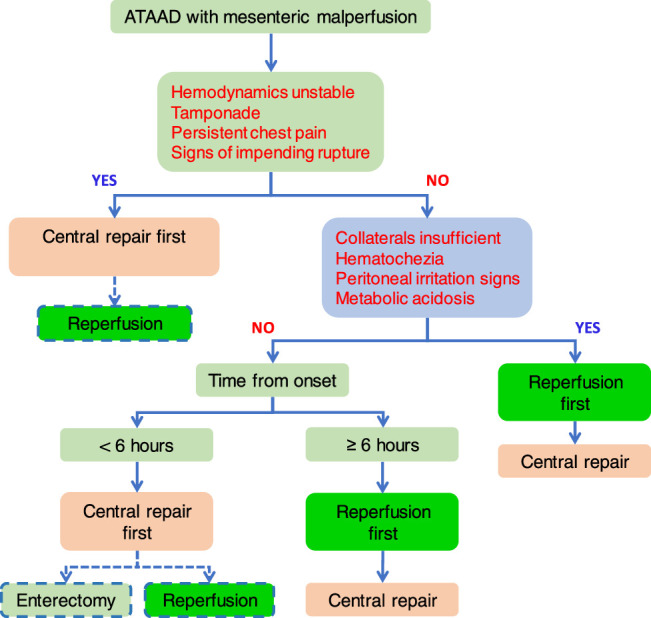
Management algorithm of acute type A aortic dissection (ATAAD) with mesenteric malperfusion. Dashed lines represent potential treatment options.

All patients were evaluated with aortic computed tomographic angiography (CTA), echocardiography, electrocardiogram, and blood tests, and received adequate analgesia and anti-impulse therapy. Management strategies were chosen based on the risk of aortic rupture and circulatory compromise, severity of bowel ischemia/necrosis, time from dissection onset (cut-off at 6 h), and mechanisms of ischemia (dynamic vs. static) (Fig. 2). Generally, reperfusion-first strategy is preferred for static ischemia and central repair-first for dynamic ischemia, as long as no moribund end-stage organ failure (coma, stroke, shock, intestinal or bowel necrosis, or hepatic failure) occurred.

1. Central repair-first is the preferred strategy for all patients with a high risk of rupture and circulatory compromise (unstable hemodynamics, or tamponade, or signs of impending rupture, or persistent chest pain, or intractable hypertension), regardless of presentations, duration and mechanisms of mesenteric ischemia;
2. Reperfusion-first strategy is indicated in patients (1) without high risk of rupture and circulatory compromise, and (2) with severe mesenteric ischemia (insufficient collaterals of SMA, or metabolic acidosis, or hematochezia, or peritoneal irritation signs), regardless of duration and mechanisms of ischemia;
3. In patients without a high risk of rupture and circulatory compromise or severe mesenteric ischemia,
  1. central repair-first is preferred strategy within 6 h from onset, and
  2. reperfusion-strategy is preferred for patients presenting 6 h or later from onset of aortic dissection.

After proximal aortic repair, arterial blood gas analysis and changes in abdominal symptoms and signs are closely monitored, and digital subtraction angiography (DSA) or CTA is used to assess SMA perfusion and assess the need for reperfusion or laparotomy. In patients with deteriorating ischemia or no signs of alleviation, the need for laparotomy or enterectomy would be evaluated with a multidisciplinary team.

### Surgical techniques

Our routine approach to ATAAD include ascending aortic and/or root repair, hemi-arch or total arch repair using a frozen elephant trunk, as has been described previously^19^. SMA angiography and stenting was performed under local or general anesthesia. The access and size of SMA stents were selected based on CTA findings and measurements. After identification of the true lumen with DSA, an Absolute Pro peripheral self-expanding bare metal stent (Abbott Cardiovascular) was inserted in the dissected SMA retrogradely or antegradely via the femoral or brachial arteries. We routinely downsize the stent by 5–10%, and in most cases the stent is 6–8 mm in diameter and 6–8 cm in length. A small bolus of contrast media would be injected before stent deployment to ensure the stent be implanted in the true lumen of SMA. A completion arteriogram would confirm the shape and patency of the SMA stent, which, if necessary, would be dilated with a balloon catheter.

### Follow-up

All hospital survivors were followed up regularly through clinic visits, phone calls, letters, emails, or social media. Aortic CTA was performed at 1 month (or discharge), 6 months, 1 year and annually henceforth to assess the aorta and changes of SMA (patency, stenosis, endoleak, etc).

### Statistical analysis

Data analysis was performed using SPSS 29 (IBM SPSS). Variables are expressed as the mean±standard deviation, or median (interquartile range, IQR), or *n* (%), and compared using the Student *t*-test, or Mann–Whitney *U* test, or Pearson χ^2^ test, or Fisher’s exact text. Survival and freedom from aortic dilatation and reoperation and SMA stent patency were estimated using Kaplan–Meier method, and intergroup comparisons were made with log-rank test. Cox regression was used to identify the risk factors for all-cause mortality. The criteria of selecting candidate variables were either factors that are of clinical relevance or have been reported frequently in literature, or those that exhibited significance in univariate analysis. Variables considered in the models included body mass index (kg/m^2^), tamponade, peritoneal irritation signs, celiac artery involvement, lactate level (mmol/l), and cardiopulmonary bypass time (minute). All tests were two-sided and a *P* value of less than 0.05 was considered statistically significant.

## Results

### Baseline characteristics

Mean age was 50.6±11.4 years (59 males. 86.8%). Dissection involved the SMA in 65 patients (95.6%), celiac artery (CA) in 41 (60.3%), left renal artery in 35 (51.5%), right renal artery in 28 (41.2%), iliac artery in 47 (69.1%), femoral artery in 8 (11.8%), and coronary arteries in 34 (50%). Abdominal manifestations included decreased or absent bowel sounds (*n*=55, 80.9%), increased or hyperactive bowel sounds (*n*=6, 8.8%), abdominal pain or distension (*n*=45, 66.2%), hematochezia (*n*=13, 19.1%), and peritoneal irritation signs (*n*=7, 10.3%). Four patients were free from abdominal symptoms (5.9%).

The two groups were otherwise similar at baseline (Table 1), except patients with the reperfusion-first strategy showed more celiac artery involvement (74.2% vs. 48.6%, *P*=0.033) and peritoneal irritation signs (19.4% vs. 2.7%, *P*=0.041), while tamponade was more common in the central repair-first group (27% vs. 3.2%, *P*=0.008).

**Table 1:** Baseline characteristics.

Variable	Whole series (*n*=68), *n* (%)	Central repair-first (*n*=37), *n* (%)	Reperfusion-first (*n*=31), *n* (%)	*P* value
Age, year	50.6±11.4	49.5±11.7	51.8±11.1	0.413
Male	59 (86.8)	32 (86.5)	27 (87.1)	0.941
BMI, kg/m^2^	26.2±3.6	26.2±4.0	26.0±3.3	0.790
Hypertension	50 (73.5)	28 (75.7)	22 (71)	0.664
Diabetes mellitus	4 (5.9)	2 (5.4)	2 (6.5)	0.856
Sleep apnea-hypopnea	17 (25)	9 (24.3)	8 (25.8)	0.889
Chronic renal failure	1 (1.5)	1 (2.7)	0	0.360
Cerebrovascular accident	6 (8.8)	4 (10.8)	2 (6.5)	0.531
Cardiovascular disease	6 (8.8)	2 (5.4)	4 (12.9)	0.281
Current or previous smoker	33 (48.5)	22 (59.5)	11 (35.5)	0.057
Alcohol use	22 (32.4)	13 (35.1)	9 (29)	0.595
Location of entry tear				0.474
Zone 0	47 (69.1)	24 (64.9)	23 (74.2)	
Zone 1	8 (11.8)	6 (16.2)	2 (6.5)	
Zone 2	7 (10.3)	3 (8.1)	4 (12.9)	
Zone 3	6 (8.8)	4 (10.8)	2 (6.5)	
Distal extent				0.880
Thoracic aorta	3 (4.4)	2 (5.4)	1 (3.2)	
Abdominal aorta	10 (14.7)	5 (13.5)	5 (16.1)	
Iliac artery	47 (69.1)	26 (70.3)	21 (67.7)	
Femoral artery	8 (11.8)	4 (10.8)	4 (12.9)	
Aortic root involvement	56 (82.4)	32 (86.5)	24 (77.4)	0.332
Coronary artery involvement				0.234
Left	3 (4.4)	1 (2.7)	2 (6.5)	
right	19 (27.9)	7 (18.9)	12 (38.7)	
left + right	12 (17.6)	7 (18.9)	5 (16.1)	
Maximal aortic diameter, mm	50.2±6.7	50.4±6.9	50.1±6.6	0.762
Maximal aortic root diameter, mm	42.0±4.8	41.5±5.0	42.7±4.6	0.287
Left ventricular (LV) ejection fraction, %	63.1±5.4	62.2±6.1	64.1±4.3	0.158
LV end-diastolic diameter, mm	47.3±6.5	47.7±6.8	46.9±6.1	0.605
Tamponade	11 (16.2)	10 (27)	1 (3.2)	0.008
Myocardial infarction	1 (1.5)	0	1 (3.2)	0.275
Superior mesenteric artery involvement	65 (95.6)	34 (91.9)	31 (100)	0.107
Celiac artery involvement	41 (60.3)	18 (48.6)	23 (74.2)	0.033
Left renal artery involvement	35 (51.5)	15 (40.5)	20 (64.5)	0.051
Right renal artery involvement	28 (41.2)	18 (48.6)	10 (32.3)	0.175
Abdominal manifestations				
Increased bowel sounds	6 (8.8)	2 (5.4)	4 (12.9)	0.281
Decreased/absent bowel sounds	55 (80.9)	31 (83.8)	24 (77.4)	0.509
Abdominal distention/pain	45 (66.2)	27 (73)	18 (58.1)	0.199
Peritoneal irritation signs	7 (10.3)	1 (2.7)	6 (19.4)	0.025
Hematochezia	13 (19.1)	7 (18.9)	6 (19.4)	0.964
Lactate, mmol/l	3.4±3.1	3.7±3.8	2.9±2.0	0.744
PH	7.35±0.06	7.35±0.07	7.35±0.05	0.224
Base excess, mmol/l	–3.21±4.27	–3.31±4.74	–3.08±3.70	0.844

Values are presented as mean±standard deviation or *n* (%).

### Surgical data

Table 2 lists the data of ATAAD repair that was performed in 64 patients, which were not significantly different between two groups. Central aortic cannulation was used in 25 (39.1%) and peripheral cannulation in 39 patients (60.9%). Unilateral antegrade cerebral perfusion was used in 58 (85.3%). Proximal aortic procedures included aortic valve resuspension in 39 patients (60.9%), Bentall/Cabrol procedure in 19 (29.7%), and David reimplantation and ascending aortic replacement in 3 patients each (4.7%). Total arch replacement with a frozen elephant trunk was performed in 59 patients (92.2%). The times of cardiopulmonary bypass, cross-clamp and circulatory arrest averaged 212±57, 125±30, and 19±8 min, respectively.

**Table 2:** Operative details.

Variables	Whole series (*n*=64), *n* (%)	Central repair-first (*n*=37), *n* (%)	Reperfusion-first (*n*=27), *n* (%)^a^	*P* value
Cannulation site				0.207
Aorta	25 (39.1)	12 (32.4)	13 (48.1)	
Peripheral artery	39 (60.9)	25 (67.6)	14 (51.9)	
Selective cerebral perfusion				0.697
Unilateral antegrade	58 (90.6)	33 (89.2)	25 (92.6)	
Bilateral antegrade	4 (6.3)	3 (8.1)	1 (3.7)	
Retrograde	2 (3.1)	1 (2.7)	1 (3.7)	
Proximal aortic procedure				0.626
Aortic valve resuspension	39 (60.9)	20 (54.1)	19 (70.4)	
Bentall/Cabrol operation	19 (29.7)	13 (35.1)	6 (22.2)	
David reimplantation	3 (4.7)	2 (5.4)	1 (3.7)	
Ascending aortic replacement	3 (4.7)	2 (5.4)	1 (3.7)	
Arch procedure				0.257
Total arch replacement	61 (95.3)	34 (91.9)	27 (100)	
Frozen elephant trunk	59 (92.2)	32 (86.5)	27 (100)	
Hemi-arch replacement	3 (4.7)	3 (8.1)	0	
Cardiopulmonary bypass time, min	212±57	208±32	216±81	0.208
Cross-clamp time, min	125±30	128±28	122±32	0.443
Circulatory arrest time, min	19±8	18±5	20±10	0.486

Values are presented as mean±standard deviation or *n* (%).

SMA, superior mesenteric artery.

^a^ In the reperfusion-first group, 3 patients died and one elected to have medical therapy after SMA stenting.

### Early mortality and morbidity

Of the whole series, early death occurred in 24 patients (35.3%), and mortality rate was significantly lower with the reperfusion-first strategy [19.3% (6/31) vs. 48.7% (18/37), *P*=0.012]. The most common causes of death were multiorgan failure, seen in 54.2% (13/24) and bowel necrosis and/or gastrointestinal bleeding, seen in 33.3% (8/24). Other causes were cerebral hernia, cardiopulmonary arrest, and aortic rupture, in 1 patient each (Table 3).

**Table 3:** Early and midterm outcomes.

Variable	Whole series (*n*=68), *n* (%)	Central repair-first (*n*=37), *n* (%)	Reperfusion-first (n=31, %)	*P* value
Early outcomes				
Early mortality	24 (35.3)	18 (48.7)	6 (19.3)	0.012
Multiple organ failure	13 (19.1)	9 (24.3)	4 (12.9)	
GI bleeding/bowel necrosis	8 (11.8)	8 (21.6)	0	
Cerebral hernia	1 (1.5)	0	1 (3.2)	
Cardiopulmonary arrest	1 (1.5)	1 (2.7)	0	
Aortic rupture	1 (1.5)	0	1 (3.2)	
Stroke	5 (7.3)	3 (8.1)	2 (6.5)	0.796
Paraplegia	3 (4.4)	1 (2.7)	2 (6.5)	0.457
Gastrointestinal complications	24 (35.3)	20 (54.1)	4 (12.9)	<0.001
Acute kidney injury	24 (35.3)	16 (43.2)	8 (25.8)	0.137
Dialysis for acute kidney injury	19 (27.9)	13 (35.1)	6 (19.4)	0.152
Reoperation for bleeding	4 (5.9)	3 (8.1)	1 (3.2)	0.398
Low cardiac output	9 (13.2)	5 (13.5)	4 (12.9)	0.941
Pneumonia	6 (8.8)	5 (13.5)	1 (3.2)	0.139
Respiratory failure	10 (14.7)	9 (24.3)	1 (3.2)	0.017
Tracheostomy	2 (2.9)	1 (2.7)	1 (3.2)	0.900
Sternal complication	1 (1.5)	1 (2.7)	0 (0)	0.360
Midterm outcomes				
Duration of follow-up, year	3.0±1.4	3.5±1.8	2.5±0.9	0.057
Late death	2 (4.5)	2 (10.5)	0	0.181
Late reoperation	0	0	0	1.000
Distal aortic dilatation	3 (6.8)	1 (5.3)	2 (8.3)	0.328
Thrombosis of SMA/celiac stent	4 (9.1)	0 (0)	4 (16)	0.121
Coronary anastomotic leak	1 (2.4)	1 (5.3)	0	0.432
Kaplan–Meier estimates at 5 years, %				
Survival	61.8 (49.2–72.2)	45.9 (29.5–60.8)	80.6 (61.9–90.8)	0.009
Freedom from late adverse events	74.7 (57.5–85.7)	76.0 (54.2–88.4)	74.9 (45.7–89.9)	0.812
Patency of SMA stent	NA	NA	84.0 (62.8–93.7)	NA

Values are presented as mean±standard deviation, or *n* (%), or Kaplan–Meier estimate (95% CI).

GI, gastrointestinal; NA, not applicable; SMA, superior mesenteric artery.

In the reperfusion-first group, following stenting of SMA, 3 died of aortic rupture while waiting for proximal aortic repair, multiorgan failure and cerebral hernia, in 1 each; another patient elected to have medical treatment and survived to the latest follow-up. ATAAD repair was performed for the 27 remaining patients at median 9.1 h after SMA stenting (IQR 1.1–78.2), 3 of whom died within 30 days.

Of the central repair-first group, 17 patients died within 30 days, and another died of septic shock outside hospital at 33 days (Fig. 1). Among 4 patients who received reperfusion after central repair, one underwent SMA stenting immediately after completion of ATAAD surgery and survived, while the three other received reperfusion at 1.75, 4 and 4 h, and succumbed to cardiopulmonary arrest and multiorgan failure at 16, 6 and 5 days, respectively.

Gastrointestinal complications and acute kidney injury were the most common morbidities, seen in 24 patients each (35.3%). Nineteen patients (27.9%) underwent dialysis for acute kidney injury. Stroke occurred in 5 patients (7.3%), paraplegia in 3 (4.4%), reoperation for bleeding in 4 (5.9%), and respiratory failure in 10 (14.7%). The two groups did not differ significantly in postoperative morbidities, except higher incidences of gastrointestinal complications (12.9% vs. 54.1%, *P*<0.001) and respiratory failure (3.2% vs. 24.3%, *P*=0.017) in the central repair-first group.

### Late outcomes

By May 2024, follow-up was complete in 100% for a mean duration of 3.3±1.4 years, which was longer in the central repair-first group (3.7±1.9 vs. 2.9±0.9 y, *P*=0.072). By the latest follow-up, no patient sustained bowel ischemia, or recurrent dissection, or thromboembolic events like stroke, or graft infection, or leakage of SMA stent, and nor did any patient require late reintervention.

#### Survival

**Figure 3:**
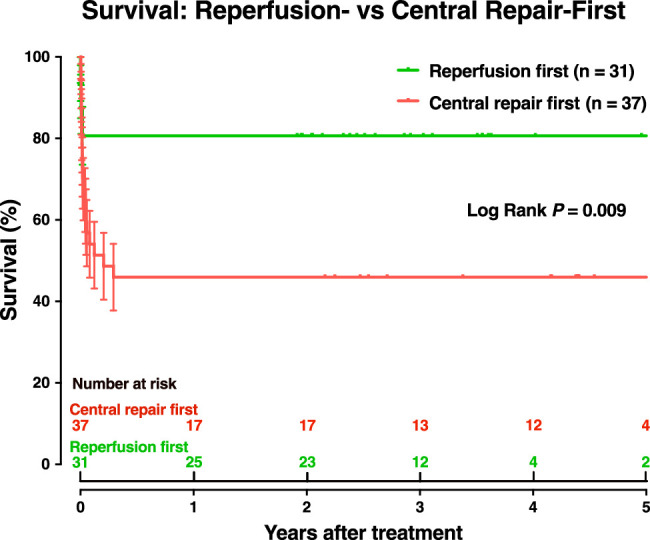
Survival was significantly higher in patients with the reperfusion-first strategy.

Two patients with central repair-first strategy died within 6 months after discharge, 1 from sepsis at 2.5 months and another from multiorgan failure at 3.5 months. For the whole series, survival was 63.2% at 3 months [95% confidence interval (CI), 50.6–73.4%] and 61.8% thereafter 61.8% thereafter (95% CI 49.2–72.1%). Patients in the reperfusion-first group showed a significantly higher survival through 5 years (80.6% vs. 45.9%, *P*=0.009) compared to central repair-first group (Fig. 3).

#### Late adverse events

Of the central repair-first group, coronary anastomotic leak and distal aortic growth (for 5 mm) were detected in 1 patient each, at 4 months and 3 years, respectively, and two reperfusion-first patients were found to have distal aortic dilatation at 16 and 13 months, respectively. All 4 patients have been asymptomatic thus far and are doing well and closely monitored.

**Figure 4:**
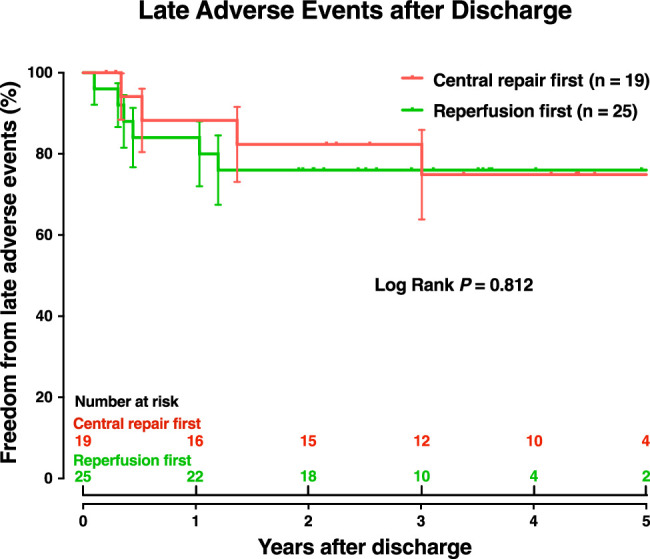
Freedom from adverse events after discharge did not differ significantly between two strategies.

Among 44 hospital survivors, freedom from late adverse events was 88.2% at 6 months (95% CI, 73.9–94.9%), 85.8% at 1 year (95% CI, 71.1–93.4%), 78.7% at 3 years (95% CI, 63.0–88.3%), and 74.7% through 5 years (95% CI, 57.5–85.7%), which did not differ significantly between the two strategies (*P*=0.812) (Fig. 4).

#### Imaging follow-up

On radiologic follow-up, available in 100% (25/25) in the reperfusion-first group, SMA stent thrombosis was detected in 3 asymptomatic patients at 33 days, 5 months, and 12 months, respectively, with 30–60% lumen stenosis but good perfusion distal to the stent. The area of the false lumen proximal to the SMA stent decreased significantly, from 51.9±28.3 mm^2^ (median 46.5, IQR, 30.7–66.0 mm^2^) preoperatively to 14.0±21.2 mm^2^ (median 0; IQR 0–26.2 mm^2^) on the latest CTA (*P*=0.002). A similarly significant shrinkage was observed in the false lumen distal to the SMA stent, from 22.6±17.3 mm^2^ (median, 19.0; IQR 11.9–32.2 mm^2^) preoperatively to 2.7±9.3 mm^2^ (median 0; IQR, 0–0) on follow-up CTA (*P*=0.003).

The estimated patency of the SMA stent was 88.0% at 6 months (95% CI, 67.3–96.0%) and 84.0% through 5 years (95% CI, 62.8–93.7%).

### Risk factor analysis

In Cox regression, tamponade [hazard ratio (HR), 3.093; 95% CI, 1.171–8.170; *P*=0.023], peritoneal irritation signs (HR, 8.559; 95% CI, 1.838–39.859; *P*=0.006), lactate level (mmol/l) (HR, 1.279; 95% CI 1.120–1.461; *P*<0.001) and cardiopulmonary bypass time (min) (HR, 1.025; 95% CI, 1.013–1.036; *P*<0.001) were risk factors for all-cause mortality, while the reperfusion-first strategy was associated with a lower risk of death (HR 0.087; 95% CI, 0.016–0.465; *P*=0.004) compared to the central repair-first strategy. Obesity index (BMI) was not identified as a risk factor for death (HR 0.343, 95% CI 0.820–1.072; *P*=0.343).

## Discussion

Management of mesenteric malperfusion in ATAAD is very difficult and challenging, and aortic repair-first vs. reperfusion-first as the optimal strategy has been a long-debated topic since the introduction of endovascular techniques to restore visceral perfusion in this extremely high-risk group of patients^10,20,21^. Despite growing experience with this highly lethal condition over the past decades, current surgical and clinical outcomes remain poor, and drawing conclusions from available data in the literature is very challenging due to the lack of an appropriate control group and the infeasibility of randomized studies^22^. In a recent comprehensive review^22^, Ayoub and associates from Duke covered the most important studies by the teams from Michigan, Stanford, Japan, and Europe^8–10,14–16,23–26^. Following a reperfusion-first strategy with various techniques, the mortality rate was lowered considerably, ranging from 0 to 25–33.9% (Table 4)^8,9,23,26^. Given the serious and continuing concern over aortic rupture before proximal repair, a reperfusion-first strategy followed immediately by central repair seems to be preferable over a delay in aortic repair. Although the Duke team went on to propose an algorithm for ATAAD with suspected mesenteric malperfusion based on existing literature and their experience, they hold that a solid clinical pathway to guide the management of such patients seems impossible to define in the light of the current meagre evidence in literature^22^.

**Table 4:** Studies on reperfusion- and central repair-first strategies for acute type A dissection with mesenteric malperfusion.

Author, year, reference	Origin	Sample size (reperfusion- vs. central repair-first)	Reperfusion technique	Duration of follow-up (and completeness)	Survival or mortality with reperfusion-first strategy
Comparative studies					
Deeb *et al*., 1997^10^	US	13 (10 vs. 3)	Fenestration, SMA stenting	1.4±1.1 years	75% survival at 1.4 years
Yamashiro *et al*., 2015^15^	Japan	10 (8 vs. 2)	right iliac to SMA bypass (vein graft)	9.6±5.2 years	100% aortic event-free survival at 5 years
Uchida *et al*., 2018^16^	Japan	12 (7 vs. 5)	Laparotomy, direct tube perfusion	Not reported	0 death in 7 with reperfusion-1st strategy
Leshnower *et al*., 2019^9^	US	31 (13 vs. 18)	TEVAR	Not reported	30% mortality in 10 patients with TEVAR-1st
Sugiyama *et al*., 2020^17^	Japan	6 (4 vs. 2)	SMA stenting	Not reported	100% late survival; 2 reinterventions at 7 and 3 months
*Subtotal*		72 (42 vs. 30)		34.7% complete (25/72) at mean 4.8±3.3 years	
Gao, 2024*	China	68 (31 vs. 37)	SMA stenting	100% complete (68/68) at mean 3.3±1.4 years	In-hospital & 30-day mortality 19.4%; 5-year survival 80.6%
Reperfusion-first only					
Midulla *et al*., 2011^14^	France	9	Fenestration	Mean 3 years	33.3% mortality at 1 month
Tsagakis *et al*., 2013^23^	Germany	12	Fenestration, SMA stenting	Not reported	25% in-hospital mortality
Yang *et al*., 2019^8^	US	82	Fenestration, SMA stenting	Not reported	39% in-hospital mortality

SMA, superior mesenteric artery; TEVAR, thoracic endovascular aortic repair.

* refers to the present study.

The University of Michigan team, who pioneered the reperfusion-first concept in 1994, has extensive experience with this strategy^8,10,13,25^. Although Yang from the same team recently reported the largest series of 82 patients managed with reperfusion-first strategy using fenestration or stenting^8^, so far only 5 studies have compared the reperfusion-first vs. central repair-first strategies in a total of 72 patients revascularized with a variety of techniques (Table 4), including fenestration, stenting, extra-anatomic or vein graft bypass, and thoracic endovascular aortic repair (TEVAR)^9,10,15–17^, and follow-up data was available in only 31.9% (23/72) of patients^15,17^. Arguably, the present study represents the largest series comparing the outcomes of two strategies for ATAAD complicated with mesenteric malperfusion.

Given the scarcity of evidence on the long-term outcomes in large series^7,10,12,16,23,27^, the results of this study imply that the reperfusion-first strategy with SMA stenting is a safe, effective and durable approach to ATAAD with mesenteric malperfusion, as proven by the reduction in early mortality by 60% (19.4% vs. 48.7%) compared to central repair-first strategy, favorable late survival (81%), freedom from adverse events (75%) and high SMA stent patency (83%) at 5 years. This study provides late outcomes data supporting the use of the reperfusion-first strategy with SMA stenting in the treatment of ATAAD with mesenteric ischemia.

The favorable outcomes with the reperfusion-first strategy may be ascribed, in part, to the technical simplicity and minimal invasiveness of SMA stenting. The whole procedure from arrival at catheter lab to stent deployment can be completed within 60 min. Much less time-consuming than open revascularization or TEVAR, it can rapidly resolve/alleviate mesenteric ischemia, immediately ending the vicious cascade of “bowel ischemia/necrosis→cardiopulmonary compromise→multiorgan failure→mortality”^10^. Ideally performed in a hybrid operating room, SMA stenting followed by proximal repair can proceed seamlessly in one single stage, addressing the aortic and mesenteric issues more rapidly and effectively.

Nevertheless, all central repair-first patients were at high risk of rupture and/or with unstable hemodynamics (coronary involvement, severe acute aortic regurgitation, tamponade), while the reperfusion-first group had relatively stable hemodynamics and low rupture risk. Although such inherent selection bias is unavoidable, the more pertinent concern is over patients between the two extremes of aorto-cardiac and visceral catastrophes, that is those with “intermediate” risk of aortic rupture, hemodynamic compromise, and bowel necrosis. Literally, the debates about central repair-first vs. reperfusion-first strategy originate from and revolve around these “intermediate” patients (Fig. 2), for whom the treatment goals should include both “preventing aortic rupture” and “salvaging the gut”. As bowel necrosis can occur as early as 6 hours from onset, while central repair can eliminate or effectively alleviate dynamic ischemia, it often takes hours, during which the ischemic bowels may become irreversibly necrotic, especially in patients presenting after 6 hours from onset. Therefore, we prefer reperfusion-first strategy for those arriving after 6 hours who may have already developed severe mesenteric ischemia. This 6-hour cut-off is also in line with the algorithm proposed by the Duke team^22^.

One patient in reperfusion-first group unfortunately died from rupture while awaiting central repair. This highlights the need to determine the optimal interval from reperfusion to central repair, and vice versa. In our experience, if DSA shows a greater than 50% increase of SMA blood flow after reperfusion, and abdominal manifestations and arterial blood gas suggest alleviation of ischemia, proximal repair should be performed as soon as possible to avoid rupture.

### Study limitations

The study has several inherent limitations, such as its retrospective nature, selection bias in treatment allocation, and lack of long-term follow-up. Particularly, the small sample size limits the statistical power for safety and efficacy concerns. Moreover, we did not include imaging analysis aimed at identifying the association of preoperative CTA findings with patient triage and treatment decision-making, and its implications for prognosis prediction. Neither does this single-center experience allow for replication of our techniques and outcomes in other institutions.

## Conclusions

In this series of ATAAD with mesenteric malperfusion, a reperfusion-first strategy using SMA stenting could significantly reduce the early mortality, and achieved favorable late survival and freedom from adverse events, with high stent patency. These results suggest that the reperfusion-first strategy may be an effective and durable approach to ATAAD with mesenteric malperfusion. Patients with tamponade, peritoneal irritation signs, elevated lactate levels and longer pump time were at added risk for all-cause mortality.

## Ethical approval

The Institutional Review Board of Henan Chest Hospital approved this retrospective study (No. 20230405).

## Consent

The Institutional Review Board of Henan Chest Hospital waived the need for individual patient consent for this retrospective study (No. 20230405).

## Source of funding

This work was supported by National Key Clinical Discipline Development Program (#2023-70) and Key Technology Research and Development Program from Henan Science and Technology Agency (#242102310254) (#2024-1463).

## Author contribution

X.G.: study concept or design; data collection; data analysis and interpretation; writing the paper; critical review and revision of the manuscript; provision of materials, patients or resources; obtaining funding; administrative, technical or logistic support; final approval of the article. Y.-X.C.: study concept or design; data collection; data analysis; drafting the first version of the manuscript. W.-G.M.: study concept or design; data analysis or interpretation; writing the paper; critical review and revision of the manuscript; statistical expertise; literature search. L.Z.: provision of materials, patients or resources; critical review and revision of the manuscript; administrative, technical or logistic support. C.C.: provision of materials, patients or resources; critical review and revision of the manuscript; administrative, technical or logistic support. P.-F.W.: provision of materials, patients or resources; critical review and revision of the manuscript; administrative, technical or logistic support. Y.-Q.Y.: study concept or design; provision of materials, patients or resources; critical review and revision of the manuscript; obtaining funding; administrative, technical or logistic support; final approval of the article.

## Conflicts of interest disclosure

The authors have no conflict of interest relevant to this work.

## Research registration unique identifying number (UIN)

Not applicable.

## Guarantor

Xia Gao; and Yi-Qiang Yuan.

## Data availability statement

We confirm that any datasets generated during and/or analyzed during the current study are available upon reasonable request to the corresponding author.

## Provenance and peer review

Not commissioned, externally peer-reviewed.
